# Eucalyptus Essential Oil Inhibits Cell Infection by SARS-CoV-2 Spike Pseudotyped Lentivirus

**DOI:** 10.3390/biomedicines12081885

**Published:** 2024-08-19

**Authors:** Sara Alonso Fernandez, Hector F. Pelaez-Prestel, Alvaro Ras-Carmona, Juan Mozas-Gutierrez, Raquel Reyes-Manzanas, Pedro A. Reche

**Affiliations:** Department of Immunology & O2, Faculty of Medicine, Complutense University of Madrid, Pza Ramon y Cajal, S/N, 28040 Madrid, Spain; saalon07@ucm.es (S.A.F.); hpelaez@ucm.es (H.F.P.-P.); aras@ucm.es (A.R.-C.); jmozas01@ucm.es (J.M.-G.)

**Keywords:** COVID-19, SARS-CoV-2, pseudovirus, eucalyptus essential oil, eucalyptol, infection inhibition assay, selectivity index, aromatherapy

## Abstract

Severe acute respiratory syndrome coronavirus 2 (SARS-CoV-2) remains a public health concern due to infections with new SARS-CoV-2 variants. Therefore, finding effective preventive and therapeutic treatments against all SARS-CoV-2 variants is of great interest. In this study, we examined the capacity of eucalyptus essential oil (EEO) and eucalyptol (EOL) to prevent SARS-CoV-2 infection, using as a model SARS-CoV-2 Spike pseudotyped lentivirus (SARS-CoV-2 pseudovirus) and 293T cells transfected with human angiotensin-converting enzyme 2 (hACE2-293T cells). First, we determined the cytotoxicity of EEO and EOL using the MTT colorimetric assay, selecting non-cytotoxic concentrations ≤ 0.1% (*v*/*v*) for further analysis. Subsequently, we evaluated the capacity of EEO and EOL in cell cultures to preclude infection of hACE2-293T cells by SARS-CoV-2 pseudovirus, using a luciferase-based assay. We found that EEO and EOL significantly reduced SARS-CoV-2 pseudovirus infection, obtaining IC_50_ values of 0.00895% and 0.0042% (*v*/*v*), respectively. Likewise, EEO and EOL also reduced infection by vesicular stomatitis virus (VSV) pseudovirus, although higher concentrations were required. Hence, EEO and EOL may be able to inhibit SARS-CoV-2 infection, at least partially, through a Spike-independent pathway, supporting the implementation of aromatherapy with these agents as a cost-effective antiviral measure.

## 1. Introduction

Infections and reinfections by severe acute respiratory syndrome coronavirus 2 (SARS-CoV-2), which caused the coronavirus disease 2019 (COVID-19) pandemic, have become frequent and the virus remains a public health concern [1,2,3]. Currently, most SARS-CoV-2 infections are mild, but severe COVID-19 cases still persist and some individuals develop long-lasting effects known as long COVID [4,5]. The severe cases arise when patients suffer a cytokine storm characterized by a sudden surge in pro-inflammatory cytokines such as IL-1, IL-6, IL-8 and TNF-α. This cytokine storm is associated with the development of acute respiratory distress syndrome (ARDS), metabolic acidosis and coagulation dysfunction, explaining most COVID-19 deaths [6,7].

SARS-CoV-2 is an enveloped virus belonging to the Coronaviridae family that primarily infects the respiratory epithelium, specifically upper and lower respiratory epithelial cells expressing angiotensin-converting enzyme 2 (ACE2) receptors [8]. ACE2 expression levels in the respiratory tract correlate with SARS-CoV-2 infection predation, with nasal ciliated cells serving as primary targets for SARS-CoV-2 replication in the early stages of infection. SARS-CoV-2 is generally contained in the upper respiratory tract, but if it reaches the lung parenchyma in the lower respiratory tract, severe disease manifests [9,10]. SARS-CoV-2 entry to ACE2-expressing cells is facilitated by the Spike protein (S protein), which is located on the virus envelope [11,12]. The S protein is a glycoprotein composed of two subunits: the receptor-binding (S1) and the cell membrane fusion (S2) subunits. The S1 contains the receptor-binding domain (RBD), which specifically binds to ACE2 [12,13,14], prompting viral entry. Given the role of the S protein in SARS-CoV-2 infection, most neutralizing antibodies (Abs) target this protein, and it is the central focus of COVID-19 vaccines [15,16].

COVID-19 vaccines have had a significant impact on COVID-19 deaths and hospitalizations [17]. However, COVID-19 vaccines have limitations: they are expensive [18], fail to provide sterile immunity [19,20] and can produce numerous adverse effects, including myocarditis and pericarditis, thrombosis and thrombocytopenia syndrome and encephalomyelitis, among others [21]. Moreover, the emergence of immune escape variants concentrating mutations in the S protein [22] challenge the immunity provided by COVID-19 vaccines and force their continuous update [16]. Thereby, there are efforts and interest in developing anti-SARS-CoV-2 drugs. Currently, various antiviral drugs have been tested in COVID-19 patients, but they have had limited success and are not widely available [23,24]. Therefore, there is an unmet demand for effective, accessible and affordable drug alternatives against COVID-19 with therapeutic and/or preventive activity [25,26]. These alternatives ought to be particularly important for low- and middle-income countries [27].

It has been proposed that natural compounds such as essential oils (EOs) may be useful as therapeutics for COVID-19 [28,29,30,31,32]. EOs are volatile secondary plant metabolites with many pharmacological properties, including antimicrobial activity [33,34]. EOs are widely available, can be easily administered and have fewer side effects than synthetic drugs [35]. Among these EOs, eucalyptus essential oil (EEO) has been highlighted as a promising anti-SARS-CoV-2 candidate [36,37].

EEO is extracted from the leaves of the eucalyptus tree and its main active constituent is eucalyptol (EOL) (1,8-cineole). Both EEO and EOL are renowned for their analgesic, antiseptic, anti-inflammatory and antimicrobial properties [33,36,38,39,40,41,42,43]. In fact, these compounds are frequently used for the treatment of respiratory tract ailments such as those from common cold, pulmonary tuberculosis, asthma, influenza, ARDS and chronic obstructive pulmonary disease (COPD), among others [38]. The specific anti-viral mechanisms of action of EEO and EOL can vary for different viruses and are largely unknown, but nonetheless they probably affect virus integrity [44].

In this work, we showed that EEO and EOL prevent pseudotyped lentivirus-expressing SARS-CoV-2 Spike protein in their membrane envelope (SARS-CoV-2 pseudovirus) from infecting 293T cells transfected with human ACE2 receptors (hACE2-293T cells). By including non-cytotoxic concentrations of EEO and EOL in infection assays, viral entry decreased, with half-maximal inhibitory concentrations (IC_50_) of 0.00895% and 0.0042% (*v*/*v*), respectively. We also found that EEO and EOL inhibition of viral entry seems to be, at least partially, Spike-independent, since these compounds also inhibited vesicular stomatitis virus pseudotyped lentivirus (VSV pseudovirus) infection. Overall, these results highlight the potential use of EEO aerosols to fight envelope viruses, including SARS-CoV-2.

## 2. Materials and Methods

### 2.1. Reagents, Cells and Plasmids

Eucalyptus essential oil (EEO) was purchased from Arganour Cosmetics (Malaga, Spain) (100% essential oil) and eucalyptol (EOL) from Sigma-Aldrich (Burlington, MA, USA) (99% eucalyptol, ref nº: C80601-100 mL). Soluble human ACE2 (hACE2) was purchased from Miltenyi Biotec (Bergisch Gladbach, Germany). 293T cells from the European Collection of Authenticated Cell Cultures were acquired from Merck KGaA (Darmstadt, Germany). Cells were tested negative for mycoplasma (Mycoplasma Gel Detection Kit, Biotools B&M Labs, S.A., Madrid, Spain) and grown in DMEM growth media, supplemented with 10% FBS, 2 mM glutamine, 100 U/mL penicillin (Lonza, Basel, Switzerland) and 100 μg/mL streptomycin (Lonza) (complete DMEM). The following plasmids for generating SARS-CoV-2 Spike pseudotyped lentivirus (SARS-CoV-2 pseudovirus) were obtained from BEI Resources (https://www.beiresources.org/ (accessed on 29 July 2021)):

- Viral entry vector encoding for the Spike glycoprotein (S) from SARS-CoV-2 strain Wuhan-Hu-1 with a D614G mutation (BEI catalog number: NR-53765)
- HIV-based lentiviral backbone vector encoding for firefly luciferase (Luc2) and synthetic Zoanthus sp. green fluorescent protein (ZsGreen1) (BEI catalog number NR-52516).
- Helper plasmids encoding for Gag and Pol (BEI catalog number NR-52517), Tat1b (BEI catalog NR-52518) and Rev1b (BEI catalog NR-52519).

The plasmid encoding the human angiotensin-converting enzyme 2 (hACE2) was purchased from Sino Biological (catalog number HG10108-CH). The plasmid encoding the glycoprotein (G protein) from the vesicular stomatitis Indiana virus (VSV) for the generation of control lentiviral particles (VSV pseudovirus) was kindly supplied by Dr. Esther M. Lafuente (Universidad Complutense de Madrid).

### 2.2. Viability Assay

The cytotoxic assays of EEO and EOL were performed on 293T cells by using the MTT (3-(4,5-dimethylthiazol-2-yl)-2,5-diphenyltetrazolium bromide tetrazolium reduction) colorimetric assay (MTT Cell proliferation kit I, Roche, Basel, Switzerland), following the manufacturer’s instructions. In brief, 293T cells were seeded per duplicate into 96-well poly-L-lysine-coated (Sigma-Aldrich, Burlington, MA, USA) plates, at 1.25 × 10^4^ cells/well in 120 μL complete DMEM and incubated at 37 °C and 5% CO_2_ overnight. The next day, EEO and EOL were tested from 0 to 1% concentration range (*v*/*v*). After 72 h, 10 μL of MTT labeling reagent was added per well and incubated for 4 h at 37 °C and 5% CO_2_. Then, 100 μL of the solubilization buffer was added and the plate was incubated overnight (37 °C and 5% CO_2_). Finally, the next day, the absorbance at 570 nm was read using a BioTek ELx800 Absorbance Microplate Reader (BioTek, Winooski, VT, USA). After the absorbance data, the percentage of dead cells (DCs) at different compound concentrations was calculated as described by Bailen et al. [45] using Equation (1):

(1) $$\%DC=\frac{\left(A_{p}-A_{b}\right)}{\left(A_{c}-A_{b}\right)}\times 100$$

where A_p_ is the absorbance at the studied condition (concentration of EEO or EOL), A_b_ the absorbance of the blank (medium) and A_c_ the absorbance of the control (culture with 0% *v*/*v* of EEO or EOL). Finally, the concentration of cytotoxicity 50% (CC_50_), the concentration needed to reduce cell viability by 50% compared to the control, of both EEO and EOL was determined after plotting the DC data using Neutcurve Python package (version 2.1.0) [46] (https://jbloomlab.github.io/neutcurve/ (accessed on 15 May 2024)).

### 2.3. Generation of Pseudovirus and hACE2-293T Cells

Pseudoviruses, encoding the S protein from SARS-CoV-2 or G protein from VSV, were obtained as described by Crawford et al. [47]. Briefly, 8 × 10^5^ 293T cells per well were seeded in a six-well plate in 2 mL of complete DMEM without penicillin and streptomycin and incubated at 37 °C and 5% CO_2_. 293T are epithelial-like cells, derived from a human kidney epithelial cell line immortalized with SV40 large T antigens, which are highly efficient in producing lentivirus particles using plasmid vectors. In addition, 293T cells can be easily transfected to express different receptors on the cell surface, allowing infection/tropism by pseudotyped lentivirus particles [48]. After a 24-h incubation, cells were co-transfected with a plasmid mix including 1 μg of HIV-based lentiviral backbone plasmid encoding the reporter protein, 0.22 μg of each of the plasmids encoding the necessary proteins for virion formation (Gag-Pol, Tat and Rev) and 0.34 μg of the plasmid enconding the entry protein, either S and the G protein, to generate SARS-CoV-2 and VSV pseudoviruses, respectively. This plasmid mix was diluted in Opti-MEM (Gibco, Waltham, MA, USA) in a final volume of 320 μL with 10 μL of Lipofectamine 2000 (Invitrogen, Waltham, MA, USA). Four hours post-transfection, the media was changed to 2 mL of complete DMEM. Cells were grown at 37 °C and 5% CO_2_. Finally, 72 h after transfection, the supernatants, containing the pseudoviruses, were harvested and filtered through a 0.45 μm SFCA low protein-binding filter (Merck KGaA, Darmstadt, Germany). Pseudoviruses were aliquoted in 200 µL samples and stored at −80 °C until use.

To generate hACE2-expressing 293T cells (hACE2-293T cells), parental 293T cells were plated in six-well plates (8 × 10^5^ cells per well) and transfected with 5 μg of the plasmid encoding hACE2 using the Lipofectamine reagent, as previously indicated. Subsequently, cells were harvested and seeded for subsequent experiments. The expression of hACE2 in transfected 293T was confirmed by flow cytometry as follows. Cells were washed with phosphate-buffered solution (PBS) (Sigma-Aldrich, Burlington, MA, USA) supplemented with 0.5% FBS and 1 mM EDTA (Merck KGaA, Darmstadt, Germany). Then, Fc receptors were blocked with 200 mg/mL IgG from human serum (Merck KGaA, Darmstadt, Germany) and cells were stained with allophycocyanin (APC)-labeled anti-hACE2 (R&D System, Minneapolis, MN, USA) diluted 1:25 in staining buffer (PBS), or with the APC-labeled Spike RBD (Miltenyi Biotec, Bergisch Gladbach, Germany) and fixed with BD CytofixTM (BD Biosciences, Franklin Lakes, NJ, USA). Data were acquired using a FACSCalibur flow cytometer (BD Biosciences, Franklin Lakes, NJ, USA) and analyzed using FlowJo software (version 10) (Tree Star, Ashland, OR, USA).

### 2.4. Titration of SARS-CoV-2 and VSV Pseudovirus

Titers of SARS-CoV-2 and VSV pseudovirus were obtained by infecting hACE2 and parental 293T cells, respectively, using the luciferase protocol described by Crawford et al. [47]. Briefly, 1.25 × 10^4^ cells per well were seeded in 96-well plates, previously coated with 50 µL of poly-l-lysine (Sigma Alrich) per well, in a final volume of 150 µL complete DMEM. For SARS-CoV-2 pseudovirus, three 1:3 serial dilutions were made starting with 50 µL of undiluted pseudovirus, and for VSV pseudovirus, two 1:50 dilutions starting with 3 µL of undiluted pseudovirus were made. Next, polybrene (Merck KGaA) was added to plates to a final concentration of 5 µg/mL by adding 7.5 µL of polybrene at 0.1 mg/mL and plates were incubated during 72 h at 37 °C and 5% CO_2_. After incubation, viral infection was detected using the Bright-Glo Luciferase Assay System (Promega, Madison, WI, USA) following the manufacturer’s instructions. In brief, 100 μL of the luciferase reagent was added to the cells and plates were incubated for 2 min at room temperature in the dark. Subsequently, cell cultures were transferred to a black-bottom plate (100 µL per well) (Thermo Fisher Scientific, Waltham, MA, USA) and relative luciferase units (RLUs) were read in an Infinite F200 Plate Reader (Tecan, Männedorf, Switzerland) with no attenuation and a luminescence integration time of 1000 milliseconds. RLUs were plotted against pseudovirus dilution. Titers of >10^6^ RLUs were achieved and an amount of virus for infection and inhibition assays of ~3 × 10^6^ RLUs per ml was selected, where there is a sufficient signal of RLUs in infected cells with regard to background (pseudovirus only) and a linear relationship between virus added and RLUs.

### 2.5. SARS-CoV-2 and VSV Pseudovirus Infection Inhibition Assay

SARS-CoV-2 and VSV pseudovirus infection of hACE2-293T or parental 293T cells, respectively, were performed in poly-L-lysine-coated 96-well plates as described earlier, but including EEO or EOL in the cell cultures. About 1.25 × 10^4^ cells in 100 μL of complete DMEM were used in all infection conditions per duplicate in 96-well plates. Pseudoviruses were diluted so that a final titer of ~3 × 10^6^ RLU per ml was reached in all infection conditions (150 µL final volume). Pseudoviruses were added to cells as previously mentioned in the Section 2.4. In the case of inhibition conditions, 30 μL of diluted pseudoviruses were first combined in a separate 96-well plate with 30 μL of complete DMEM with 36 μg/mL of hACE2 (inhibition control), or EEO and EOL at different concentrations. Next, 50 μL of these diluted pseudoviruses were added to cells. Final concentrations in cells of hACE2 and EEO/Eucalyptol were 6 µg/mL and 0.00055%-0.1% (*v*/*v*), respectively. To obtain a maximum infectivity condition, cells were cultured with diluted pseudoviruses alone (50 µL pseudoviruses and 100 μL complete DMEM). A background control (BC) condition for SARS-CoV-2 pseudovirus infection was obtained by infecting parental 293T cells with SARS-CoV-2 pseudoviruses, and also by including conditions consisting of pseudoviruses (both SARS-CoV-2 and VSV pseudovirus) without cells (100 µL of complete DMEM plus 50 µL diluted pseudoviruses). To facilitate viral infections, polybrene was added to plates to a final concentration of 5 µg/mL per well as indicated above, and plates were incubated for 72 h at 37 °C and 5% CO_2_. Viral infections were then detected using the luciferase-based assay system, and the fraction infectivity (FI) per condition was determined using the following Equation (2):

(2) $${FI}_{x}=\frac{{RLU}_{x}-{RLU}_{b}}{{RLU}_{m}-{RLU}_{b}}$$

where RLU_x_ is the luminescence reading for a specific condition, RLU_b_ is the luminescence reading of the background control and RLU_m_ is the luminescence reading of the maximum infectivity (MI) (pseudovirus with just hACE2-293T cells). The BC consisted of a condition including either only pseudovirus (infections with VSV pseudovirus) or pseudovirus with parental 293T cells (infections with SARS-CoV-2 pseudovirus). The half-maximal inhibitory concentration (IC_50_) of EEO and EOL was computed after plotting the FI data by non-linear regression using Neutcurve Python package (version 2.1.0): https://jbloomlab.github.io/neutcurve/ (accessed on 15 May 2024) [46]. Finally, a selectivity index (SI) was determined using Equation (3):

(3) $$SI=\frac{{CC}_{50}}{{IC}_{50}}$$

### 2.6. Fluorescence Microscopy

SARS-CoV-2 pseudovirus infections were visualized via fluorescence microscopy. hACE2-293T cells were infected with SARS-CoV-2 pseudovirus, as described above, without or with EOL (0.03% *v*/*v* final concentration). After 72 h of culture, pictures of five random fields were taken at 100× magnification with a microscope Floid Cell Imaging Station (Life Technologies, Carlsbad, CA, USA), and the number of cells per field was counted with Fiji, version 2.15.1 (https://github.com/fiji/fiji, (accessed on 10 February 2024)).

### 2.7. Statistics

Mann–Whitney U tests for independent samples were applied to assess statistically significant differences between infected cells in treated and non-treated samples from microscopy images. Krustal–Wallis tests were used for comparing FI differences concerning concentrations of EEO and EOL. A *p*-value < 0.05 was considered significant. Statistical analyses were carried out using GraphPad Prism, version 8.0.2 (Dotmatics, Boston, MA, USA).

## 3. Results

### 3.1. Cytotoxicity of EEO and EOL

**Figure 1:**
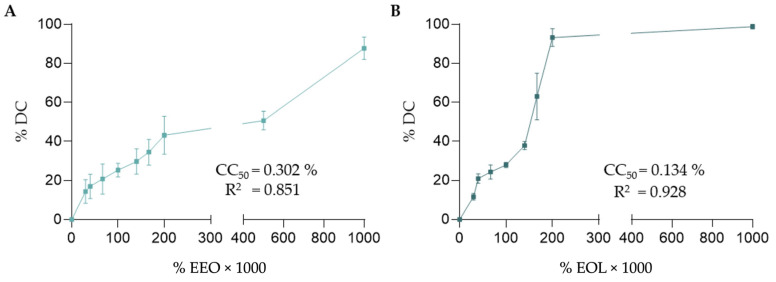
In vitro cytotoxic activity of EEO and EOL. 293T cells were incubated during 72 h with varying concentrations of EEO and EOL (0–1% *v*/*v*). Cell viability was assessed with the MTT assay and the percentage of DC calculated as described in the Materials and Methods. Graphs depict the percentage of DC at the tested concentrations of EEO (Panel **A**) and EOL (Panel **B**). In the plots, concentrations of EEO and EOL are in percentage (% *v*/*v*) and multiplied by 1000. Experiments were carried out 5 times and mean values with error bars corresponding to SEM are represented. CC_50_ of EEO and EOL values shown in the graphs were computed after a non-linear regression using Neutcurve Python package (version 2.1.0).

Given that eucalyptus essential oil (EEO) and eucalyptol (EOL) can be toxic, we first examined the cytotoxicity of these compounds on 293T cells that were used in infection inhibition assays. To that end, we cultured 293T cells for 72 h in the presence of varying concentrations of EEO and EOL ranging from 0 to 1% (*v*/*v*), and cell viability/death was assessed using the MTT assay, determining the percentage of death cells (DCs) at the different concentrations of the compounds (details in Section 2). As shown in Figure 1, EOL was more cytotoxic than EEO. For example, the percentage of DC reached about 40% with 0.2% (*v*/*v*) of EEO, while with the same concentration of EOL, the percentage of DC was over 90%. Furthermore, the concentration needed to produce 50% of cell mortality (CC_50_) of EEO and EOL was 0.302% and 0.134% (*v*/*v*), respectively. Following these results, we selected a working concentration of EEO and EOL ≤ 0.1% (*v*/*v*) for inhibition assays.

### 3.2. Inhibition of SARS-CoV-2 Pseudovirus Infections by EEO and EOL

**Figure 2:**
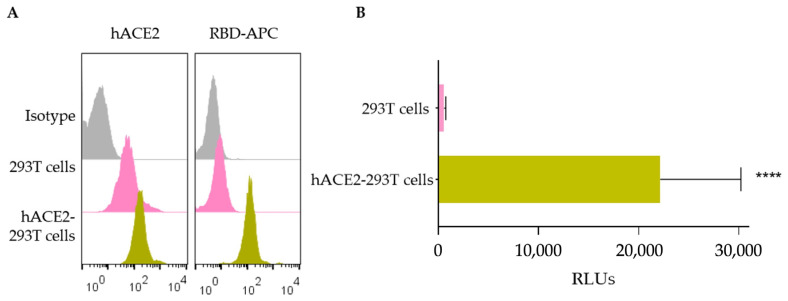
Generation of hACE2-293T cells and infection with SARS-CoV-2 pseudovirus. (**A**) ACE2 expression in hACE2-293T cells was confirmed by flow cytometry. Parental 293T cells were transfected with the plasmid encoding hACE2 and 72 h after transfection, cells were collected, stained with an anti-hACE2 antibody and analyzed by flow cytometry (details in Section 2). Histograms depict the expression of ACE2 in transfected and untransfected 293T cells using APC anti-hACE2 antibody (**left**) and APC-RBD (**right**); (**B**) SARS-CoV-2 pseudovirus infection. 293T and hACE2-293T cells were infected with SARS-CoV-2 pseudovirus (~3 × 106 RLUs) and infection was detected by luciferase-based assay system after 72 h. The graph represents relative luciferase units (RLUs) (*X*-axis) in both hACE2-293T and 293T cell lines (*Y*-axis). Experiments were carried out 13 times and mean values with error bars corresponding to SEM are represented. Mann–Whitney U test was carried out to compare significant differences between infected cells in hACE2-293T and 293T cell lines. “****” notes statistically significant differences with *p* < 0.0001.

We obtained SARS-CoV-2 Spike pseudotyped lentivirus (SARS-CoV-2 pseudovirus) as detailed in Material and Methods. SARS-CoV-2 pseudoviruses are engineered to express S protein from SARS-CoV-2 in their envelope so that they can infect cells displaying the human receptor ACE2 (hACE2). Hence, we first obtained 293T cells expressing hACE2 (hACE2-293T cells) (Figure 2A) and verified that only hACE2-293T cells could be infected by SARS-CoV-2 pseudovirus (Figure 2B). hACE2-293T cells were infected using a pseudovirus concentration of ~3 × 10^6^ RLUs per ml which was selected upon titration assays (details in Section 2).

**Figure 3:**
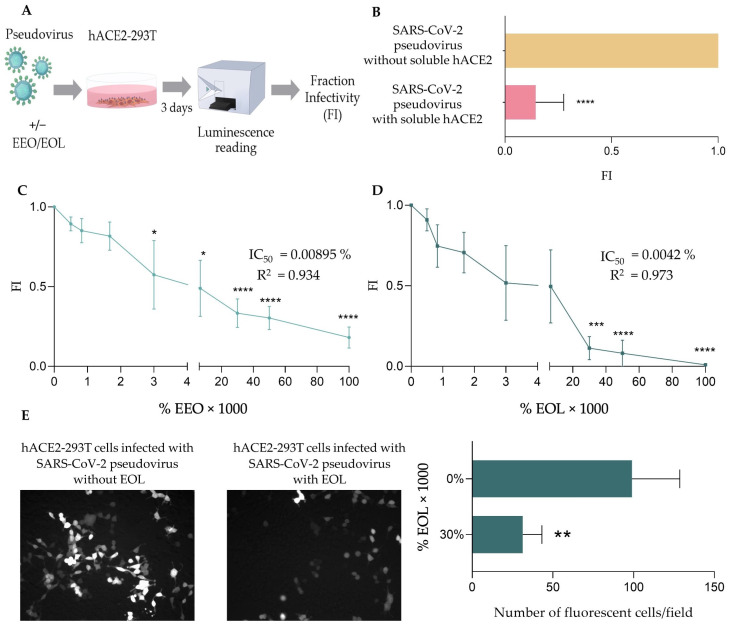
Inhibition of SARS-CoV-2 pseudovirus with EEO and EOL. (**A**) Experimental strategy. hACE2-293T cells were incubated during 3 days with SARS-CoV-2 pseudoviruses (~3 × 10^6^ RLUs per mL) in combination with varying non-toxic concentrations of EEO and EOL (0–1% *v*/*v*). Subsequently, infection was detected using a luciferase-based assay system and the % of infected cells (Fraction Infectivity) was obtained. (**B**) Inhibition of infection by soluble hACE2. As a control, hACE2-293T cells were infected with SARS-CoV-2 pseudoviruses, previously combined with soluble hACE2 (6 µg/mL final concentration). Fraction infectivity (*X*-axis) with or without hACE2 (*Y*-axis) is plotted. Experiments were carried out 11 times and mean values with error bars corresponding to SEM are represented. Mann–Whitney U test was carried out to compare significant differences between infected hACE2-293T cells with SARS-CoV-2 pseudovirus conjugated with or without soluble hACE2. “****” notes statistically significant differences with *p* < 0.0001. (**C**) and (**D**) Inhibition by EEO and EOL, respectively. Fraction infectivity (*Y*-axis) at different concentrations of EEO or EOL (*X*-axis) is plotted. In the plots, concentrations of EEO and EOL are in percentage (% *v*/*v*) and multiplied by 1000. Experiments were carried out 7 times and mean values with error bars corresponding to SEM are represented. Statistically significant differences were obtained by applying Kruskal–Wallis tests where “*”, “***” and “****” note statistical differences with regard to the maximum fraction infectivity with *p* < 0.05, *p* < 0.001 or *p* < 0.0001, respectively. IC_50_ of EEO and EOL values shown in the graphs were obtained after non-linear regression using the Python package Neutcurve (version 2.1.0) (**E**) Visualization of SARS-CoV-2 pseudovirus infection. hACE2-293T cells were infected with SARS-CoV-2 pseudovirus (~3 × 106 RLUs per mL) in the absence or presence of EOL (0.03% *v*/*v*) and after 72 h of infection were visualized by fluorescence microscopy imaging. The left panels correspond to representative microscopy images of hACE2-293T cells in the noted conditions. The graph on the right shows the number of fluorescent cells counted per field in the noted conditions with or without EOL. Mean values with error bars corresponding to SEM are represented. Mann–Whitney U test was carried out to compare significant differences between infected cells in treated and non-treated samples. Significant difference *p* < 0.01 is noted as “**”.

To test the ability of EEO and EOL to inhibit the infection by SARS-CoV-2 pseudovirus, we followed the experimental strategy depicted in Figure 3A. Briefly, we incubated hACE2-293T cells with SARS-CoV-2 pseudoviruses combined with varying concentrations of EEO or EOL (0–0.1% *v*/*v* final volume concentration) and after 3 days of incubation, we determined the fraction of cells that became infected (FI) as described in the Materials and Methods. As a control, we verified that infection could be inhibited using soluble hACE2 (Figure 3B). We found that both EEO and EOL impaired infection by SARS-CoV-2 pseudovirus at very low concentrations (Figure 3C,D), but EOL was more effective at the highest concentrations. Thus, the concentrations of EEO and EOL required to reduce the FI by 50% (IC_50_) were similar, 0.00895 and 0.0042% (*v*/*v*), respectively. However, a concentration of EOL of 0.033% (*v*/*v*) yielded an FI value of 10%, similar to that obtained in the presence of soluble hACE2 (Figure 3B), while the FI at the same concentration of EEO was over 30%.

The inhibition of SARS-CoV-2 pseudovirus infection by EOL was also verified by fluorescence microscopy. To that end, hACE2-293T cells were infected with SARS-CoV-2 pseudovirus as described in Figure 3A with and without EOL (0.03% *v*/*v*) and infection was analyzed using fluorescence microscopy, taking images of five random fields (details in Section 2). As shown in Figure 3E, infection of hACE2-293T cells by SARS-CoV-2 pseudovirus is notably handicapped in the presence of EOL.

We also tested the capacity of EEO and EOL to inhibit the infection by lentiviral particles of the vesicular stomatitis Indiana virus (VSV pseudovirus), as previously described, but using 293T cells instead of hACE2-293T cells. We found that EEO and EOL also inhibited the infection by VSV pseudovirus, but higher concentrations, particularly of EEO, were required in comparison with SARS-CoV-2 pseudovirus (see Supplementary Figure S1). Thus, the IC_50_ of EEO and EOL obtained in VSV pseudovirus was 0.069% and 0.0089% (*v*/*v*), respectively.

## 4. Discussion

Respiratory viruses are notoriously resilient to vaccines, and SARS-CoV-2 is not an exception [49]. Indeed, despite mass vaccinations, SARS-CoV-2 infections remain high and still pose an important public health concern [3]. Thereby, the search for anti-SARS-CoV-2 drugs with prophylactic and/or therapeutic properties is of great value. Various commercially available antiviral drugs, such as ribavirin, lopinavir, ritonavir and remdesivir, have been tested in COVID-19 patients, but their benefits have been either negligible and/or disputable [50,51,52,53]. Moreover, these antiviral drugs may have unforeseen adverse effects and are expensive, which limits their accessibility in low-income countries. Approved COVID-19 drugs and COVID-19 drugs undergoing late stage clinical trials have been extensively reviewed by Feng and Fu [54], concluding that no current drugs are effective in protecting against COVID-19, although ongoing new trials may provide more positive results. Hence, the availability of effective and affordable anti-SARS-CoV-2 treatments remains an unmet medical need [25,26,27]. In this context, natural products such as essential oils (EOs) with known antiviral and anti-inflammatory effects may be useful against COVID-19 [32,36]. Indeed, Paidi et al. [55] have reported that eugenol, a component extracted from clove and holy basil essential oils, can inhibit infection by SARS-CoV-2 pseudovirus in vitro, likely by blocking the interaction between the S1 protein and the hACE2 of host cells. Similarly, here we show that Eucalyptus essential oil (EEO) and its major component, eucalyptol (EOL), inhibited SARS-CoV-2 pseudovirus infection of hACE2 transfected 293T cells (hACE2-293T cells) very efficiently (Figure 3). It is worth noting that hACE2-293T cells used in infection assays were not sorted for hACE2 expression after transfection. Although such sorting would have increased the number of cells susceptible to infection, we found it unnecessary since (A) the transfection efficiency of 293T cells with hACE2 was very high, (B) expression of hACE2 was readily detected on transfected cells (Figure 2A), (C) untransfected cells did not get infected (Figure 2B) and (D) we worked with fraction infectivity (FI) values that were relativized to the condition in which all hACE2-expressing cells could get infected (Equation (2)).

In this experimental context, we found that both components, EEO and EOL, inhibited the SARS-CoV-2 infection of hACE2 transfected cells with an IC_50_ of 0.00895% and 0.0042% (*v*/*v*), respectively. These concentrations were well below the concentrations at which EEO and EOL displayed cytotoxicity (CC_50_ of 0.302% and 0.134% *v*/*v*, respectively). EEO and EOL cytotoxicity was specifically determined in 293T cells since it has been reported that it can vary between different cell lines [56,57]. After these values, we determined that the selectivity index (SI) of EEO and EOL in SARS-CoV-2 pseudovirus infection was over 33.74 and 31.9, respectively (Table 1). The SI is the ratio between the CC_50_ and IC_50_, which is used to determine the potential pharmaceutical utility of a drug. The higher the SI, the safer and more effective a drug can be considered. More specifically, it is generally accepted that a compound with a SI > 10 is a promising pharmaceutical drug [58]. In this context, as observed in Table 1, EEO and EOL can be considered promising antiviral drugs. Interestingly, we also found that EEO and EOL could inhibit infection by VSV pseudovirus, but to a lesser extent (Table 1), with a SI value of 4.38 and 15.06, respectively. These results suggest that the antiviral activity of EEO and EOL in inhibiting viral entry might be Spike/ACE2-independent. Nonetheless, EEO and EOL interactions with Spike and/or ACE2 cannot be totally ruled out and deserve to be investigated.

EEO and EOL inhibition of viral entry may be due to a direct effect on the viruses, compromising their integrity and/or structure, for example by altering the viral envelope. EEO and EOL contain lipophilic molecules that could easily penetrate the viral envelope and change their dynamic properties, as described for related compounds [44,59]. This possibility, rather than a disadvantage, amplifies the antiviral spectrum of EEO and EOL to any envelope virus. In fact, it has also been reported that EEO and/or EOL can effectively reduce infections by different envelope viruses, including herpes simplex [60,61,62,63,64,65] and influenza [66,67] viruses. Another possibility is that these components might mask viral proteins essential for the absorption and/or entry of the virus into host cells [59]. In addition, we cannot discard that EEO or any of its components, including EOL, may have alternative antiviral properties that may be more specific against SARS-CoV-2. Tulbah et al. [68] have shown that eucalyptol nano-emulsion formulations can decrease SARS-CoV-2 particles produced in vitro in Vero cells by altering viral replication and viral adsorption. Moreover, Sharma and Kaur [69], as well as Panikar et al. [70], showed an in silico study in which EOL could inhibit M^pro^, the main viral protease of SARS-CoV-2.

Besides the aforementioned antiviral properties, EEO and EOL stand out for their anti-inflammatory properties [40,41,42,43]. Indeed, EEO is commonly used to relieve airway inflammation symptoms from respiratory tract infections [71]. Moreover, EOL has been shown to protect from influenza virus infections in mouse models, likely by reducing inflammation [72,73]. Likewise, EEO and/or EOL could be useful to palliate/prevent acute respiratory distress in COVID-19 patients and other effects of the cytokine storm, as already suggested [37].

Given all this evidence, the adoption of EEO aromatherapy in crowded places (e.g., public transportation), hospitals and nursing homes would be a reasonable and appropriate measure. EEO aromatherapy can be implemented through various methods, including air nebulization, which can be easily deployed anywhere at low cost [74]. Moreover, circumstantial clues support the effectiveness of adopting EEO aromatherapy. EEO is a volatile compound released by *Eucalyptus* trees, which are particularly abundant in countries like Australia and Indonesia. Interestingly, these countries have had very few cases of COVID-19. However, we are not aware of any epidemiological study linking eucalyptus tree forests/plantations with the low incidence of COVID-19, although it is a fine hypothesis worth studying. Likewise, it has been noted that populations with high consumption of EEO infusions had fewer cases of SARS-CoV-2 [75]. Nevertheless, further epidemiological data and case studies will be necessary to determine the effectiveness of adopting EEO aromatherapy as an antiviral measure.

EEO aromatherapy is considered safe and we are unaware of any adverse effects or contraindications. However, as with any treatment or therapy, there could unforeseen adverse effects that need to be investigated, particularly in vulnerable populations. Moreover, EEO poisoning could result from accidental consumption of EEO used in EEO aromatherapy [76]. Interestingly, EEO aromatherapy can have additional and unsuspected benefits such as enhancing cognitive function, as noted in pilot studies carried out in nursing homes [77].

## 5. Conclusions

EEO and EOL inhibited in vitro cell infection by SARS-CoV-2 Spike pseudotyped lentivirus with a high selectivity index, supporting aromatherapy with these agents as a cost-effective antiviral and anti-COVID-19 measure. Geriatric homes and healthcare settings will be the major beneficiaries of EEO or EOL aromatherapy. However, clinical and epidemiological studies are required to evaluate the benefits of implementing EEO or EOL aromatherapy, as well as potential unforeseen adverse effects.

## Figures and Tables

**Table 1:** Inhibition of SARS-CoV-2 and VSV pseudovirus infections by EEO and EOL.

Pseudovirus	Compound	^a^ CC_50_ (*v*/*v*%)	^b^ IC_50_ (*v*/*v*%)	^c^ SI
SARS-CoV-2	EEO	0.302	0.00895	33.74
	EOL	0.134	0.0042	31.9
VSV	EEO	0.302	0.069	4.38
	EOL	0.134	0.0089	15.06

^a^ CC_50_: concentration needed to reduce cell viability by 50%; ^b^ IC_50_: concentration needed to reduce viral infectivity by 50%; ^c^ SI: ratio between CC_50_ and IC_50_.

## Data Availability

The original contributions presented in the study are included in the article/Supplementary Material, further inquiries can be directed to the corresponding authors.

## Supplementary Materials

The following supporting information can be downloaded at https://www.mdpi.com/article/10.3390/biomedicines12081885/s1, Figure S1: Inhibition of VSV pseudovirus infection by EEO and EOL.
