# Supplementary material for: Eucalyptus Essential Oil Inhibits Cell Infection by SARS-CoV-2 Spike Pseudotyped Lentivirus
Source: Biomedicines. 2024 Aug 19;12(8):1885. doi: 10.3390/biomedicines12081885 (PMC11351113; doi:10.3390/biomedicines12081885)
Supplement: Supplementary file 1 [file biomedicines-12-01885-s001.zip › biomedicines-3139805-supplementary.pdf]

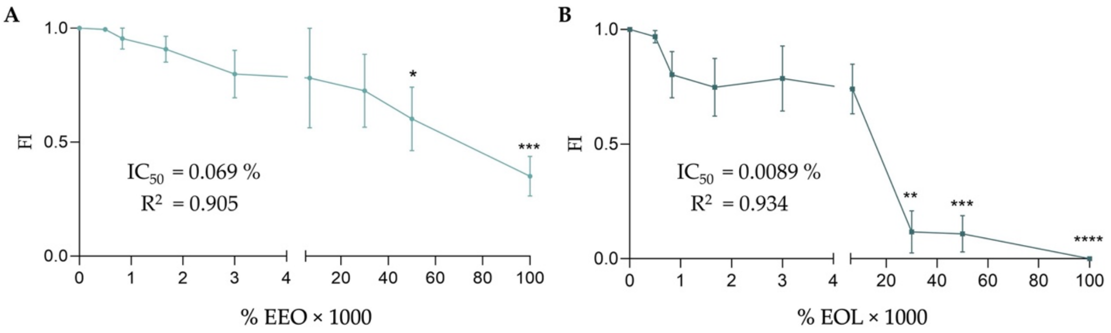

Figure S1. Inhibition of VSV pseudovirus infection by EEO and EOL. 293T cells were infected with VSV pseudoviruses in the presence of EEO or EOL (0-0.1% v/v) and infection was detected by the luciferase-based assay system. Fraction infectivity in the presence of EOO and EOL is plotted in panels A and B, respectively. Experiments were carried out 5 times and mean values with error bars corresponding to SEM are represented. Statistically significant differences were obtained by applying Kruskal-Wallis tests. Significant differences ($p < 0.05$, $p < 0.01$, $p < 0.001$ or $p < 0.0001$) with regard to the maximum infectivity (without EEO or EOL) are noted as "*", "**", "***", and "****", respectively. IC50 of EEO and EOL values shown in the graphs were obtained after a non-linear regression using the neutcurve Python package (version 2.1.0).
